# Computer simulation of cervical tissue response to a hydraulic dilator device

**DOI:** 10.1186/1742-4682-10-64

**Published:** 2013-11-06

**Authors:** Nenad Filipovic, Dalibor Nikolic, Igor Saveljic, Irena Tanaskovic, Nebojsa Zdravkovic, Aleksandar Zivanovic, Petar Arsenijevic, Branislav Jeremic, Slobodan Arsenijevic

**Affiliations:** 1 Faculty of Engineering, University of Kragujevac, Kragujevac 34000, Serbia; 2 Faculty of Medical Sciences, University of Kragujevac, Kragujevac 34000, Serbia

**Keywords:** Cervix dilation, Hydraulic balloon dilator, Finite element simulation

## Abstract

**Background:**

Classical mechanical dilators for cervical dilation are associated with various complications, such as uterine perforation, cervical laceration, infections and intraperitoneal hemorrhage. A new medical device called continuous controllable balloon dilator (CCBD) was constructed to make a significant reduction in all of the side effects of traditional mechanical dilation.

**Method:**

In this study we investigated numerically the cervical canal tissue response for Hegar and CCBD using our poroelastic finite element model and in-house software development. Boundary conditions for pressure loading on the tissue for both dilators in vivo were measured experimentally. Material properties of the cervical tissue were fitted with experimental in vivo data of pressure and fluid volume or balloon size.

**Results:**

Obtained results for effective stresses inside the cervical tissue clearly showed higher stresses for Hegar dilator during dilation in comparison with our CCBD.

**Conclusion:**

This study opens a new avenue for the implementation of CCBD device instead of mechanical dilators to prevent cervical injury during cervical dilation.

## Introduction

Cervical dilation is used not only for childbirth but also for diagnostic and therapeutic procedures [1,2]. Mechanical dilation is characterized by an increase of the cervical diameter until dilation procedure in completed. The use of mechanical dilator induces significant forces, which could damage cervical tissue and affect the fertility [3,4] or cause complications [2]. Several attempts have been made to reduce the force for cervical dilation by using pharmacological agents, which, however, can cause bleeding and cramping prior to the surgical procedure [5]. In order to avoid damage of cervical tissue, it is important to understand the structure and biomechanical behavior of this complex tissue.

Cervical tissue consists of less than 15% of smooth muscle cells and an extracellular matrix (ECM) rich in collagen [6]. The biomechanical strength of connective tissue is determined by the collagen concentration of collagen types (predominantly types I and III, IV) [7,8], the proteoglycans decorin and biglycan which affect collagen fibrillogenesis [9,10], the amount and types of collagen cross-links [11,12], the orientation of collagen fibers [13] and the concentration of elastin and water [14].

While the anatomy of cervical tissue is known, it is important to note that biomechanical models are not widely examined. A nonlinear response of cervical tissue in vivo conditions is observed but not quantified. Ex vivo analysis was used to quantify mechanical properties of the cervix [15]. Several finite element studies with anisotropic visco-hyperelastic of female pelvic modeling were described in [16-18]. To our knowledge, there is no literature data for finite element studies on cervical dilation.

In our previous pilot study [19] we introduced a continuous controllable balloon dilator (CCBD) [20] in order to achieve a smoother mechanical cervical dilation, as well as a significant reduction of the side effects observed when traditional mechanical dilation is applied [21]. Also, we presented a unique system of in vivo measurement which can determine the pressure which acts directly on cervical tissue.

In this study we analysed numerically effective wall stress response from cervical tissue and compared the results from traditional Hegar and hydraulic CCBD where boundary conditions for pressure are measured from in vivo patient data. We analysed the cervix as a porous hydrated soft tissue with a simplified geometrical tube deformable model. The innovative part of this study is the comparison of traditional Hegar and hydraulic CCBD using a computational porous model for cervical tissue which we developed.

## Methods

### CCBD

**Figure 1:**
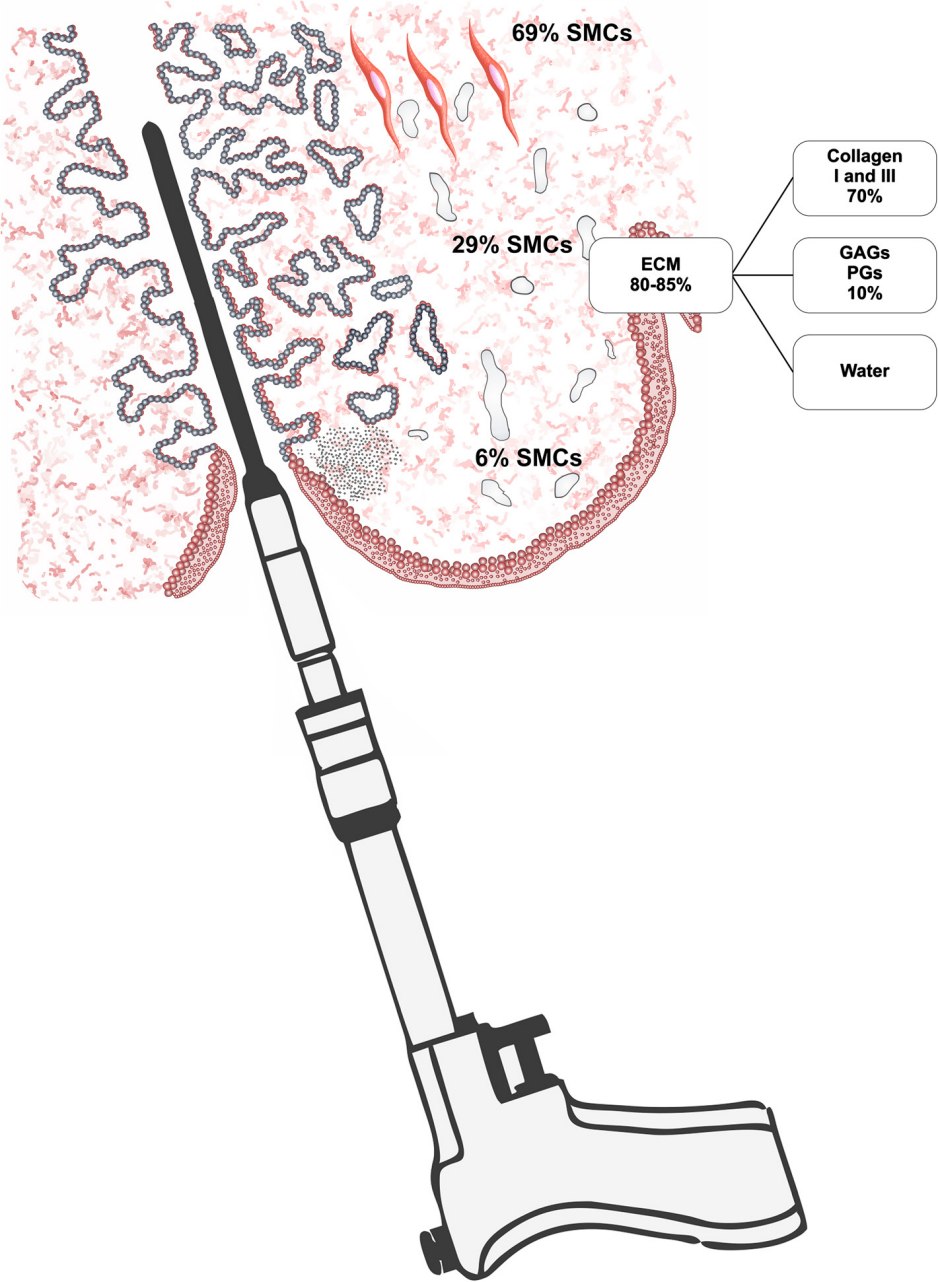
CCBD and main constituens of human cervical extracellular matrix.

The CCBD is a fully controllable device for cervical dilation. It uses a specially constructed balloon dilator that consists of three layers: an inner silicone layer, a central layer made from high-strength fabric, and an outer silicone layer. The outer silicone layer is in contact with cervical tissues during dilation. The maximum pressure of 25 bars was detected with no risk for breakage. The practical reliability of the CCBD was confirmed *in vitro* and *in vivo*[19]. The study (ISRCTN54007498) was conducted at the Gynecology & Obstetrics Clinics at Kragujevac Clinical Center, Serbia, and Podgorica Clinical Center, Montenegro. The data were collected by the coordinators of the study at the participating centers. The protocol was approved by each participating center’s institutional review board. In Figure 1 the CCBD and the main constituents of human extracellular matrix are presented. In particular, according to the literature data, approximately 80-85% of the cervix consists of an extracellular matrix (ECM) [22]. ECM of cervical stroma is composed of thick collagen fibers responsible for the tensile strength, very small amounts of elastin imparts elasticity and amorphous ground substance composed principally of glycosaminoglycans, proteoglycans and water which contributes to the integrity of the tissue [15,22].

### Numerical procedure

Cervical tissue is considered to be a porous deformable media [23]. We implemented finite a element formulation where the nodal variables are: displacements of solid, **u**; fluid pressure, **p**; Darcy’s velocity, **q**; A standard procedure of integration over the element volume is performed and the Gauss theorem is employed. An implicit time integration scheme is implemented and the balance equations are satisfied at the end of each time step. The system of differential equations which is solved for each finite element is:

(1) muu00000mqu00u¨¯t+Δtp¨¯t+Δtq¨¯t+Δt+00cuqcpucpp000cqqu˙¯t+Δtp˙¯t+Δtq˙¯t+Δt+kuukup000kpq0kqpkqqΔu¯Δp¯Δq¯=fut+Δtfpt+Δtfqt+Δt

where terms with **m** denote the mass matrix, terms with **c** denote damping, terms with **k** denote stiffness matrix, terms with **f** denote force vector for full dynamics system of displacements, pressures and fluid velocities equations. More details about all variables in eq. (1) are given in [23].

The above equations are further assembled and the resulting FE system of equations is integrated incrementally, with time step Δ*t*, transforming this system into a system of algebraic equations. A Newmark integration method is implemented for the time integration.

We analyzed the dynamic response of cervical canal. An imposed loading pressure on cervical tissue elicits an effective stress. Our model assumes formulation of a small deformation. The corresponding material constants in finite element model are modulus of elasticity E and permeability coefficient k. These material constants were fitted by standard least square method and the obtained values are E = 0.15 MPa , k = 3●10^-15^ m^4^/Ns [15]. Geometry model represents a simple cervical canal as a porous tube which is inflated. Boundary conditions are prescribed uniform pressures along the cervical canal tissue for both dilators in the zone of dilator-tissue contact. Time step used for simulation was Δt = 0.1 s which is enough to track dynamical changes during dilation process over 1 minute [23].

## Results

**Figure 2:**
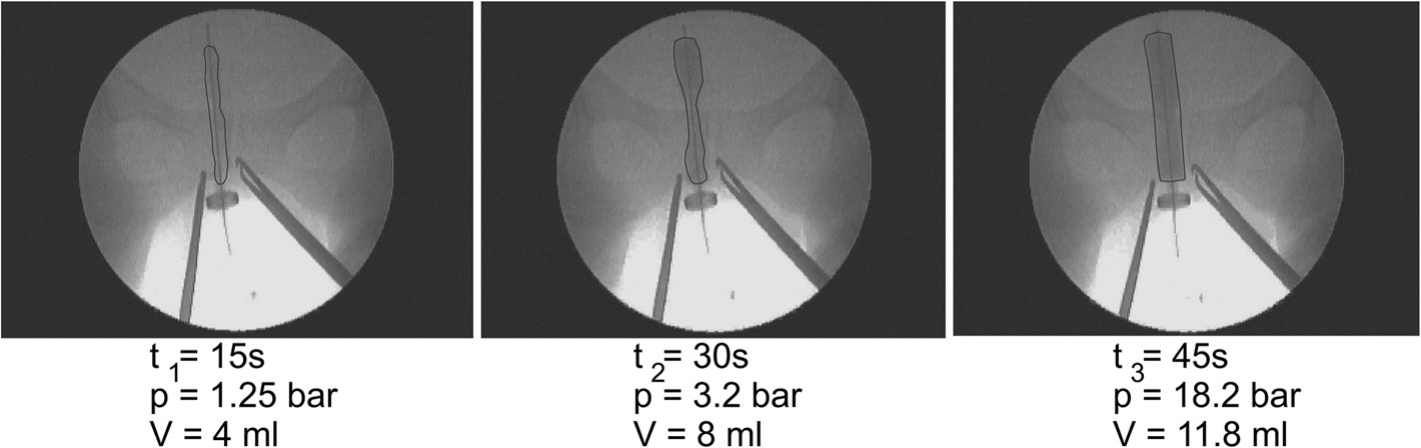
**Ballon shape during dilation process of cervix canal in-vivo for different time.** Pressure and volume of dilation for CCBD in time.

The change in the ballon shape during the dilation process of cervical canal in vivo using the CCBD is shown in Figure 2 for different time points. The pressure and volume of fluid inside the CCBD are indicated in three different time steps: 20 sec, 40 sec, 60 sec. Volume of the balloon is used to fit the finite element poroelastic model with the total radial opening of the cervix canal.

**Figure 3:**
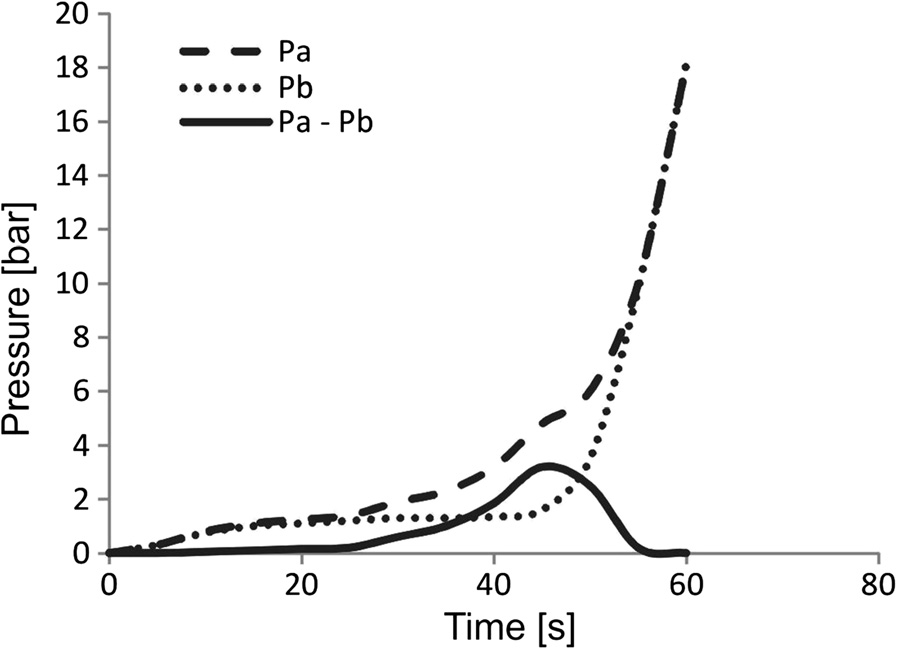
Pressure measurement in-vitro (Pa), in vivo (Pb) and total pressure from dilator to the cervical canal tissue (Pa-Pb).

The CCBD dilation procedure involves inserting ballon dilator in its initial form into the cervical canal, which results in a very low resistance to penetration. The dilation was performed synchronously along the entire length of the cervical canal, where the relative movement between the tissue/balloon dilator contact pair was reduced to almost zero [19]. If we subtract the pressure measurement from in vitro (Pa) and in vivo (Pb) during CCBD procedure, the total pressure loading on the cervical canal can be calculated, which is illustrated in Figure 3 with solid line (Pa-Pb).

**Figure 4:**
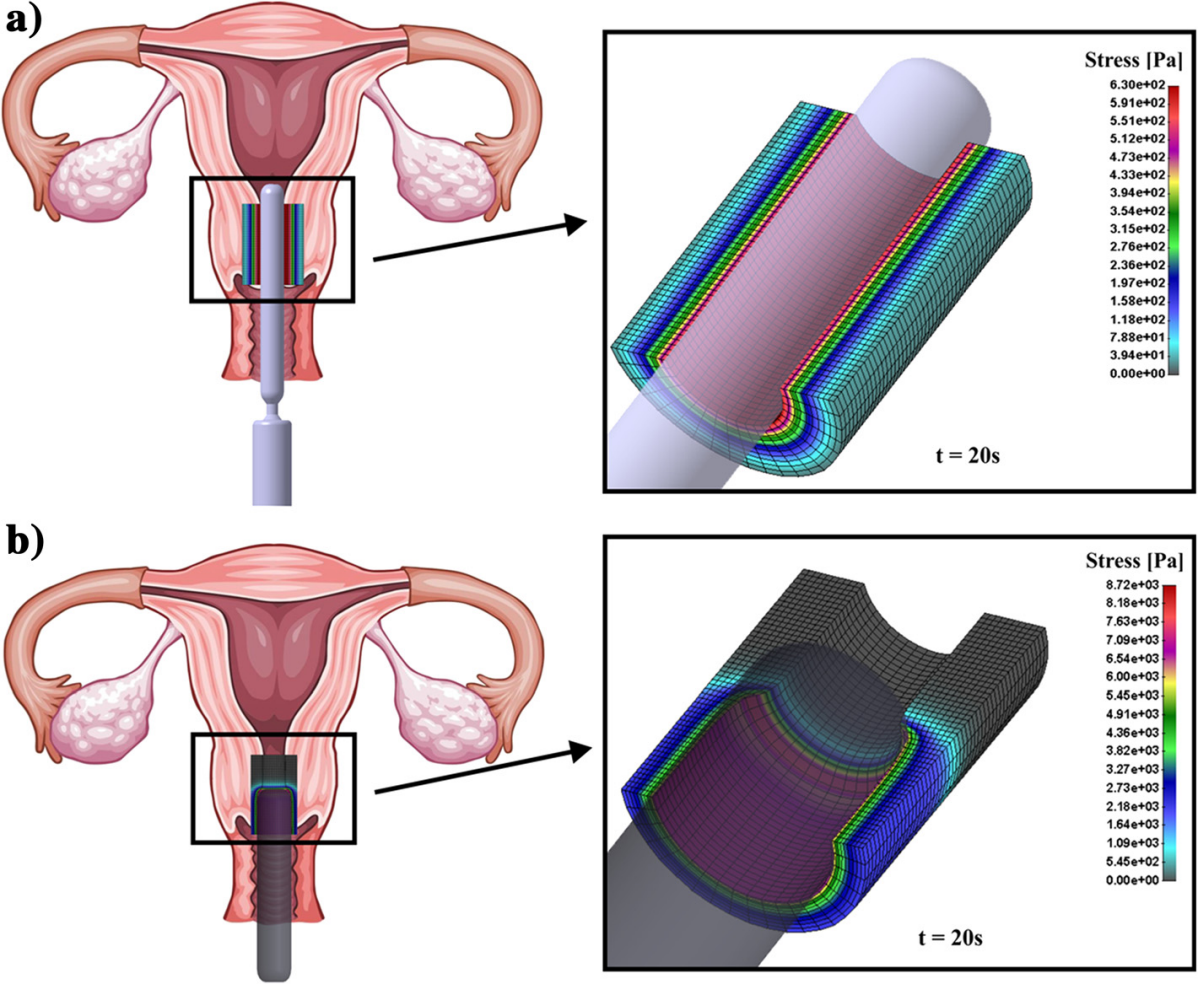
**Computational model of cervical canal.** The part which is zoomed presents the tissue in cylindrical shape where the boundary condition is the pressure loading from dilator; **a)** Case with CCBD; **b)** Case with Hegar dilator.

The basic difference between classical Hegar and CCBD is that the CCBD was initially positioned along the entire length of the cervical canal, while Hegar mechanically opened the canal with high resistance of the tissue (Figure 4a,b). The part which is zoomed in Figure 4a,b represents the cervical canal having a cylindrical shape. Plane symmetry has been considered and only half of the model has been calculated.

**Figure 5:**
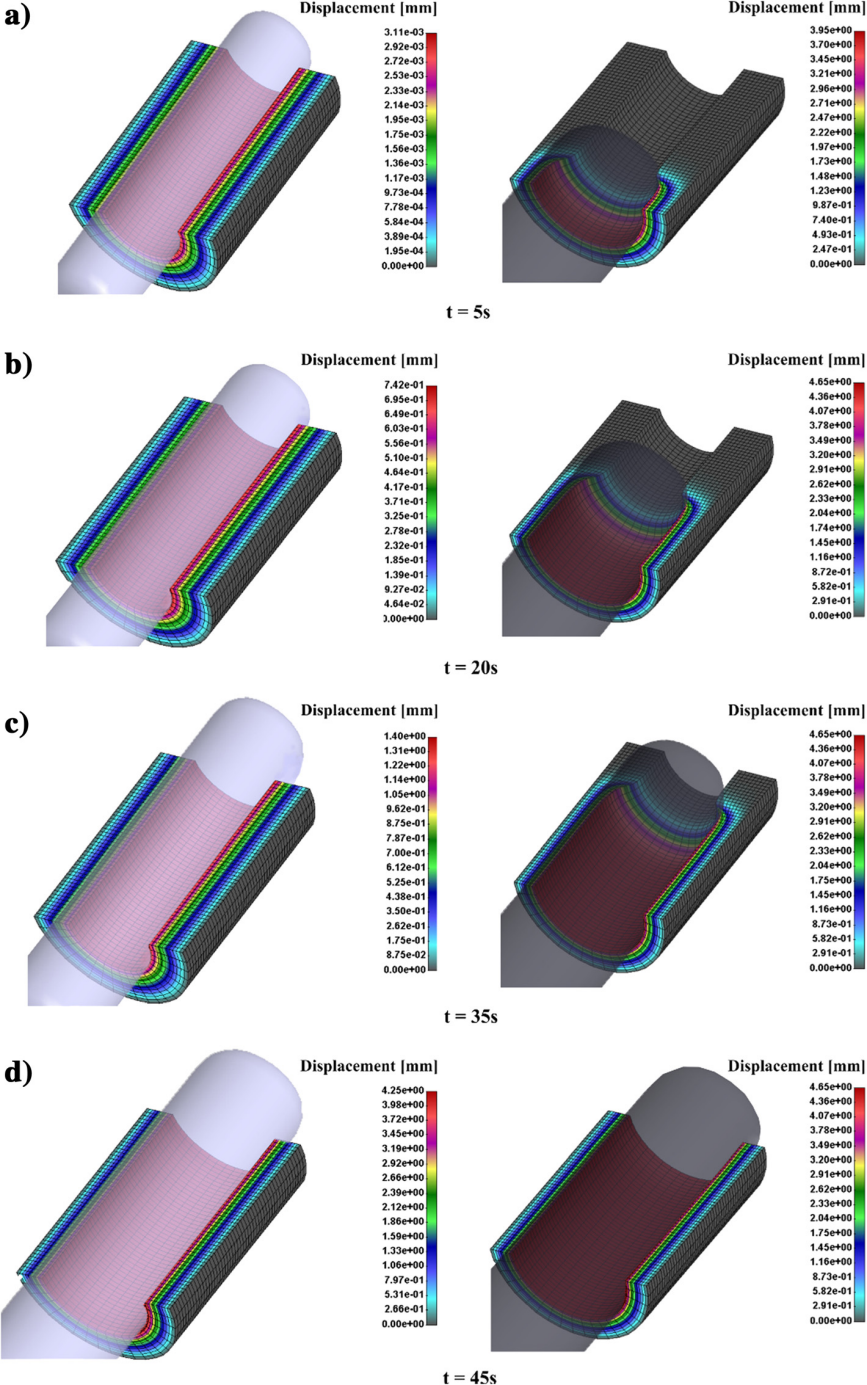
Displacement radial distribution of cervical tissue canal for CCBD (left panel) and Hegar dilator (right panel) in time; a) Displacement after 5 sec; b) Displacement after 20 sec; c) Displacement after 35 sec; d) Displacement after 45 sec.

We compared the displacement radial distribution of cervical canal for CCBD and Hegar dilator in time; 5 sec, 20 sec, 35 sec and 45 sec. Obviously Hegar dilator produces a higher radial displacement in the cervical tissue because it has a constant diameter of 8 mm and CCBD was continually opened with hydraulic pump until the final position at 45 sec (Figure 5).

**Figure 6:**
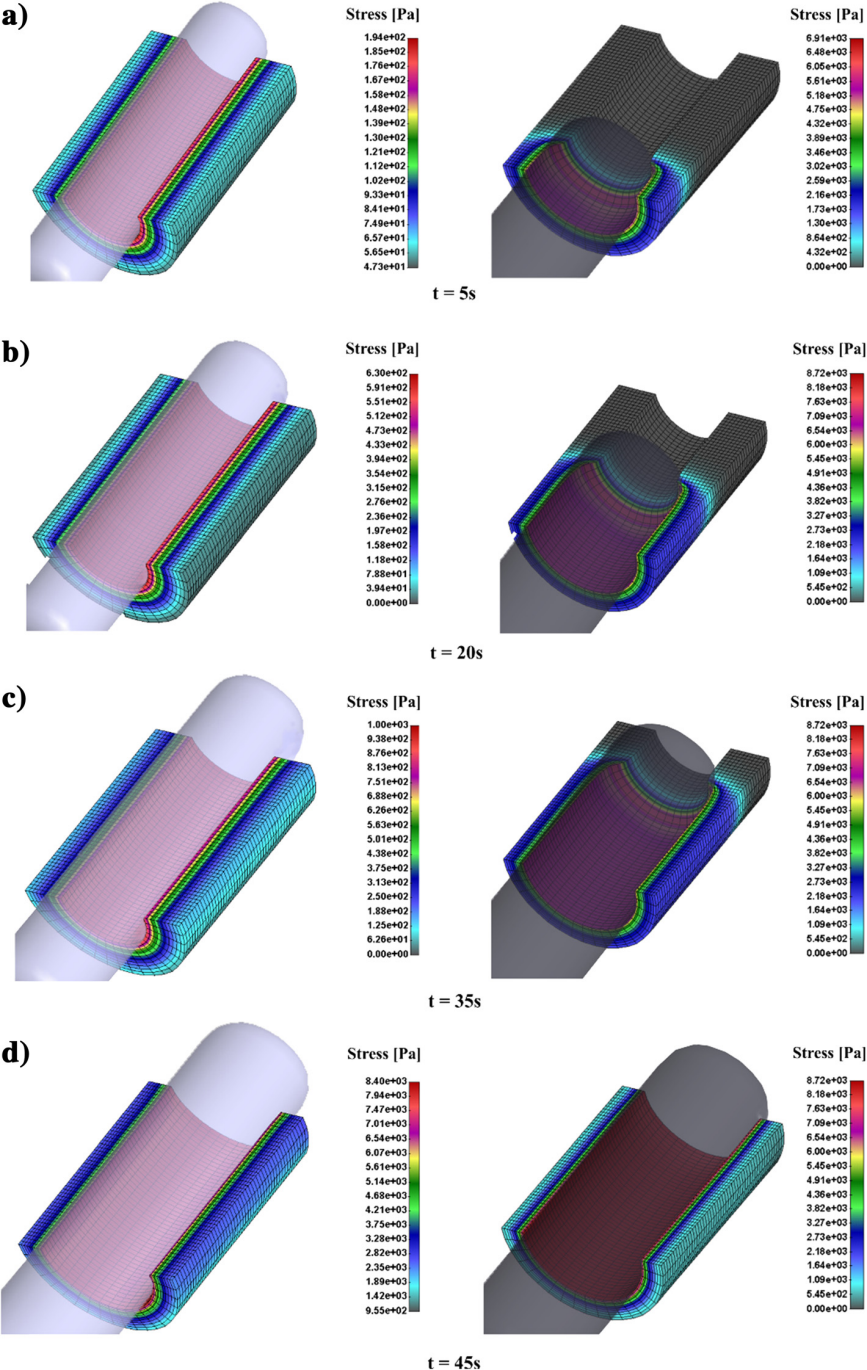
**Effective stress distribution for cervical tissue canal for CCBD (left panel) and Hegar dilator (right panel) in time. a)** Effective stress after 5 sec; **b)** Effective stress after 20 sec; **c)** Effective stress after 35 sec; **d)** Effective stress after 45 sec.

Effective stress distribution for cervical tissue canal for CCBD and Hegar dilator in time is presented in Figure 6. It can be observed that CCBD induces smaller effective stress in cervical tissue. For example, after 35 sec Hegar dilator produces 8.7 kPa while CCBD produces 1.0 kPa which is almost nine times lower. At the end of the dilation process within time frame of 45 sec the effective stress becomes similar in both dilators, which is reasonable because of the similar diameter at that time point.

Basic difference between Hegar and CCBD is a total flexibility for CCBD during the opening of the cervical canal. Displacement results clearly show a different radial opening of the cervical canal for Hegar and CCBDs. A very low resistance to penetration for CCBD could reduce damage of cervical tissue. Measurement of the pressure during CCBD process with precise pressure control on the cervical canal gives far more opportunities for future dilation procedure.

## Conclusions

Effective stress inside cervical tissue during the dilation procedure in vivo is not possible to be measured. There are some in vitro measurements which investigate separately the cervical tissue sample. Obviously, CCBD induces a continuous radial displacement position with reduced effective stress during the dilation process. Computational simulations can give insight into this complex dilation procedures and open new avenues for implementing the CCBD device in the current medical practice.

## Competing interests

The authors declare that they have no competing interests.

## Authors’ contributions

NF proposed the computer simulation model and comparison between Hegar and CCBD. Furthermore, NF processed and analyzed the obtained data, prepared all the illustrations and a major part of the manuscript. DN, IS, NZ participated in the implementation of the computer model and its simulations. IT contributed to histological images and description of cervical tissue. AZ, PA and SA substantively contributed to this work from a clinical point of view and clinical study. BJ is one of the main designers of the CCBD device. BJ contributed to the device description and clinical measurements of the dilation process. SA gave the final approval of the version to be published. In addition, all the authors contributed to this work, with numerous valuable ideas and proposals. All the authors read and approved the final manuscript.
